# COVID-19 Pandemic: Adaptation in Antenatal Care for Better Pregnancy Outcomes

**DOI:** 10.3389/fgwh.2020.599327

**Published:** 2020-11-13

**Authors:** Peace Uwambaye, Gerard Nyiringango, Sandra Marie Grace Musabwasoni, Ali Husain, Kamrun Nessa, Mohammed S. Razzaque

**Affiliations:** ^1^Department of Preventive & Community Dentistry, School of Dentistry, University of Rwanda College of Medicine and Health Sciences, Kigali, Rwanda; ^2^Department of Nursing, School of Nursing and Midwifery, University of Rwanda College of Medicine and Health Sciences, Kigali, Rwanda; ^3^Department of Midwifery, School of Nursing and Midwifery, University of Rwanda College of Medicine and Health Sciences, Kigali, Rwanda; ^4^Department of Pathology, Lake Erie College of Osteopathic Medicine, Erie, PA, United States; ^5^Department of Obstetrics & Gynecology, Chittagong Medical College Hospital, Chittagong, Bangladesh

**Keywords:** COVID-19, community health worker, pregnancy, antenatal care, safe delivery

## Introduction

Pregnancy is a precious time for an expectant mother, full of excitement and anticipation. Pregnant women need to be aware of various events of pregnancy, including how the fetus will develop and grow in the maternal womb (1). Pregnant women are usually curious about their expected due date of delivery, the recommendations regarding nutrition and exercise, and information related to the safety of the unborn baby. Good pregnancy-related care is paramount for the health of an expectant mother and the normal development of the fetus. Pregnancy is also the time to promote healthy behaviors and good parenting skills. Though pregnancy itself is not a disorder, some undesirable changes may occur during pregnancy due to an altered physiological state, such as nausea, vomiting, edema, varicose veins, heartburn, constipation, backache, tiredness, loss of sleep, hypertension, diabetes, and abnormal bleeding (2–4). Presently, there is not enough information to know whether pregnant women have a higher risk of COVID-19-related illness, although pregnant women are at greater risk of non-COVID-19-associated respiratory infections (5–9). Also, the potential risk of COVID-19 positivity during pregnancy on maternal and fetal health needs carefully designed studies. Preliminary observations, however, suggested premature birth to pregnant women with COVID-19 (10, 11). Some studies also reported infants born to mothers with COVID-19 tested positive for COVID-19, even though the virus was not present in the amniotic fluid or placenta (12). There have been reports of neonates testing positive for COVID-19 30 h after birth, confirming that transmission was not intrauterine. Currently, there is little to no evidence in the literature about the vertical transmission of COVID-19 from mother to fetus. Two studies aimed to detect SARS-CoV-2 in amniotic fluid both reported that no antibody against the virus was detected in women who were pregnant at the time, again suggesting that intrauterine transmission had not occurred (13, 14). COVID-19 presents similar pathogenesis to the SARS virus with a low risk of vertical transmission (13). The close relationship between SARS-CoV-2 and SARS can help predict that the risk of vertical transmission from mother to child is low, and further clinical studies would validate such assumption. Further research will shed light on the impact of the virus on mother and fetus during pregnancy and after delivery.

**Figure 1:**
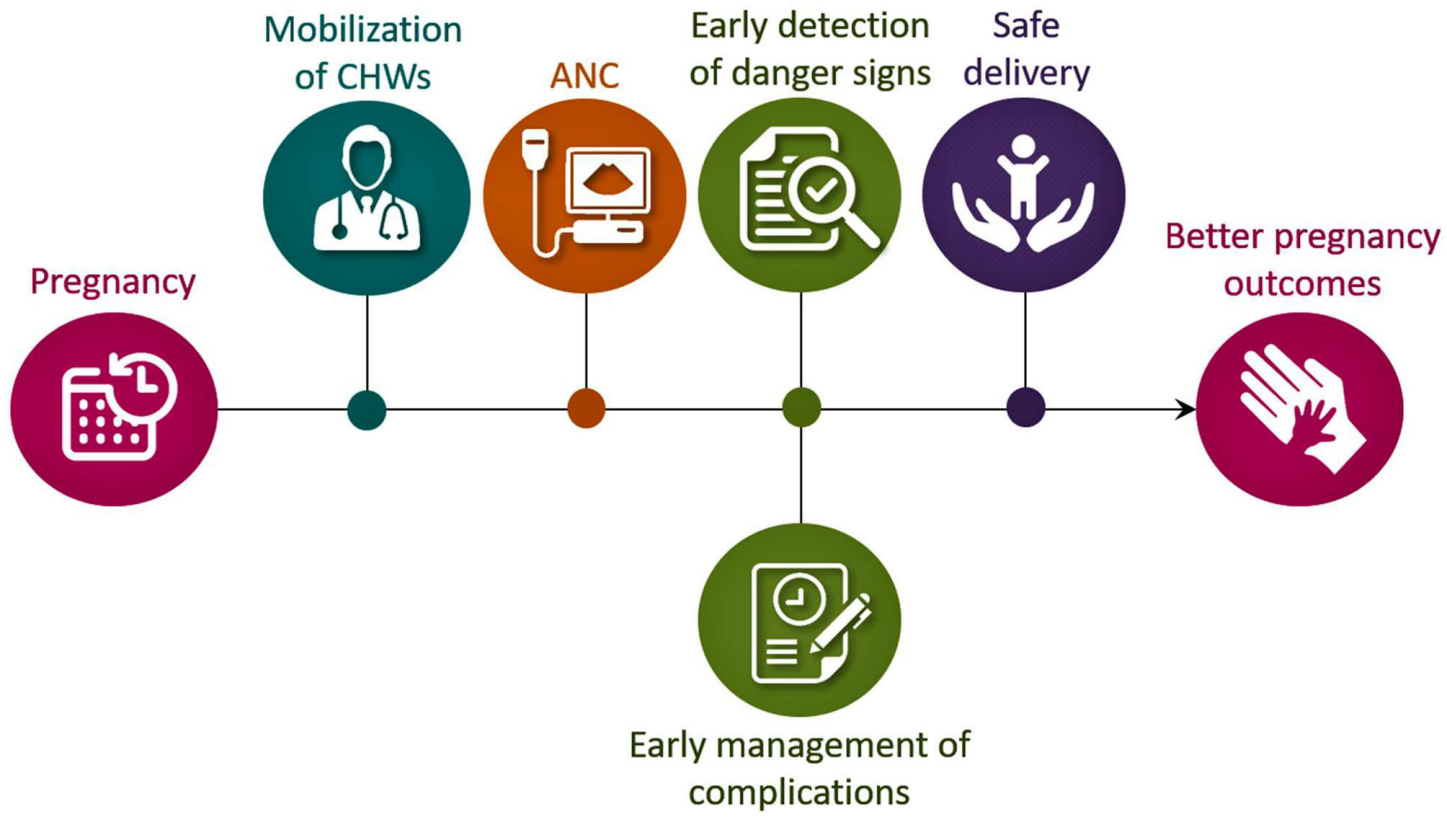
Schematic diagram showing the importance of CHWs and ANC for better pregnancy outcomes, particularly in developing countries. CHWs, community health workers; ANC, antenatal care.

Regular consultation with a health professional is recommended throughout the pregnancy, known as antenatal care (ANC) visits (1, 15–17). ANC is a critical opportunity for healthcare providers to deliver necessary support and educate pregnant women on unexpected events. As mentioned, effective ANC visits are essential for both maternal and fetal health. The ANC visits help to promote a healthy lifestyle, that include informing patients about sources of good nutrition, detecting and treating any preexisting diseases, counseling, and supporting women who may be encountering domestic violence. The World Health Organization (WHO) provided guidelines for ANC visits, including clinical examination to rule out severe anemia (hemoglobin test), detection of symptomatic sexually transmitted diseases (rapid syphilis test) and their treatment, urine test (multiple dipsticks), blood group and rhesus status, obstetrical examination (like symphysis-fundal height, presentation and position of the fetus, liquor amount, fetal heart rate), vaginal examination (where necessary), monitoring vital signs and parameters (blood pressure, maternal weight/height), and tetanus toxoid vaccination (17). Moreover, during ANC, women are advised to take iron and folic acid supplementation, which is vital for maternal and fetal health. Similarly, it is during ANC visits that pregnant women are educated on emergency danger signs of pregnancy-related complications and given the instructions for delivery and recommendations for lactation and contraception. In developing countries, ANC also increases the chance of using a skilled attendant or community health workers (CHWs) at birth to minimize maternal and fetal health risks (Figure 1). Furthermore, pregnant women need to know some diseases that can affect pregnancy outcomes, such as APH, pre-eclampsia, eclampsia, anemia, diabetes, and malaria (in malaria-endemic zones like sub-Saharan African countries). When these diseases are not adequately treated, they can lead to serious complications that impact both maternal and fetal health. Studies have suggested that optimal pregnancy outcomes of a diabetic pregnant woman rely heavily on the quality of diabetes management before and during pregnancy (18–23); such practice goes beyond diabetes, and include all other systemic diseases that might influence normal maternal and fetal evolvement during pregnancy.

It is of utmost importance to the pregnant women to get in touch with the ANC providers if they encounter COVID-19 symptoms or if they are exposed to people with COVID-19; the confirmatory test is recommended for the virus that is causing COVID-19. Pregnant women with COVID-19-positivity should be treated for fever, pain, or coughing; in more severe illness, hospitalization should be recommended. Also, for the pregnant women with COVID-19, the induction of labor or a caesarian section delivery might need additional screening extra precaution before entering the labor and/or delivery unit. Due to concern that newborns might be infected with COVID-19, infants born to COVID-19 positive mothers would need to be temporarily separated (12). Little is known about the vertical transmission in women with COVID-19 to the newborns; studies, however, noted that viral pneumonia in pregnant women is associated with an increased risk of preterm birth, fetal growth restriction (FGR), and perinatal mortality (24). Consequently, women with COVID-19 during pregnancy may present with high fever due to pneumonia, though there is no clear evidence that SARS-CoV-2 undergoes intrauterine or transplacental transmission.

Khan et al. (9) noted in their study that three pregnant women with COVID-19 did not find any vertical transmission. Among the three studied cases, one was preterm, which was not due to vertical transmission, and perhaps related to pneumonia and psychological stress during pregnancy. No evidence of maternal to the neonatal intrapartum transmission of COVID-19 was noted (9). This study was echoed by other studies that also documented no evidence for intra-uterine vertical transmission of COVID-19 from infected pregnant mothers to their fetuses (25, 26). However, precautions to prevent the spread of infection and early treatment when pregnant women get infected should always be a priority. Although it is unclear whether the virus causing the COVID-19 can be spread through breast milk, an infected mother is likely to transmit the virus, perhaps by respiratory droplets during breast-feeding; pumping out breast milk with proper precautions might be one of the safer options. During breast-feeding the mother should wear a mask and gloves. Coovadia et al. (27) reported that mothers who exclusively breastfed reduced the chances of transmitting HIV to the child compared to mothers who did not exclusively breastfeed (replacement or mixed feeding) (27). Further studies will explain whether a similar phenomenon of protection through breast milk could be achieved for mothers with COVID-19.

**Figure 2:**
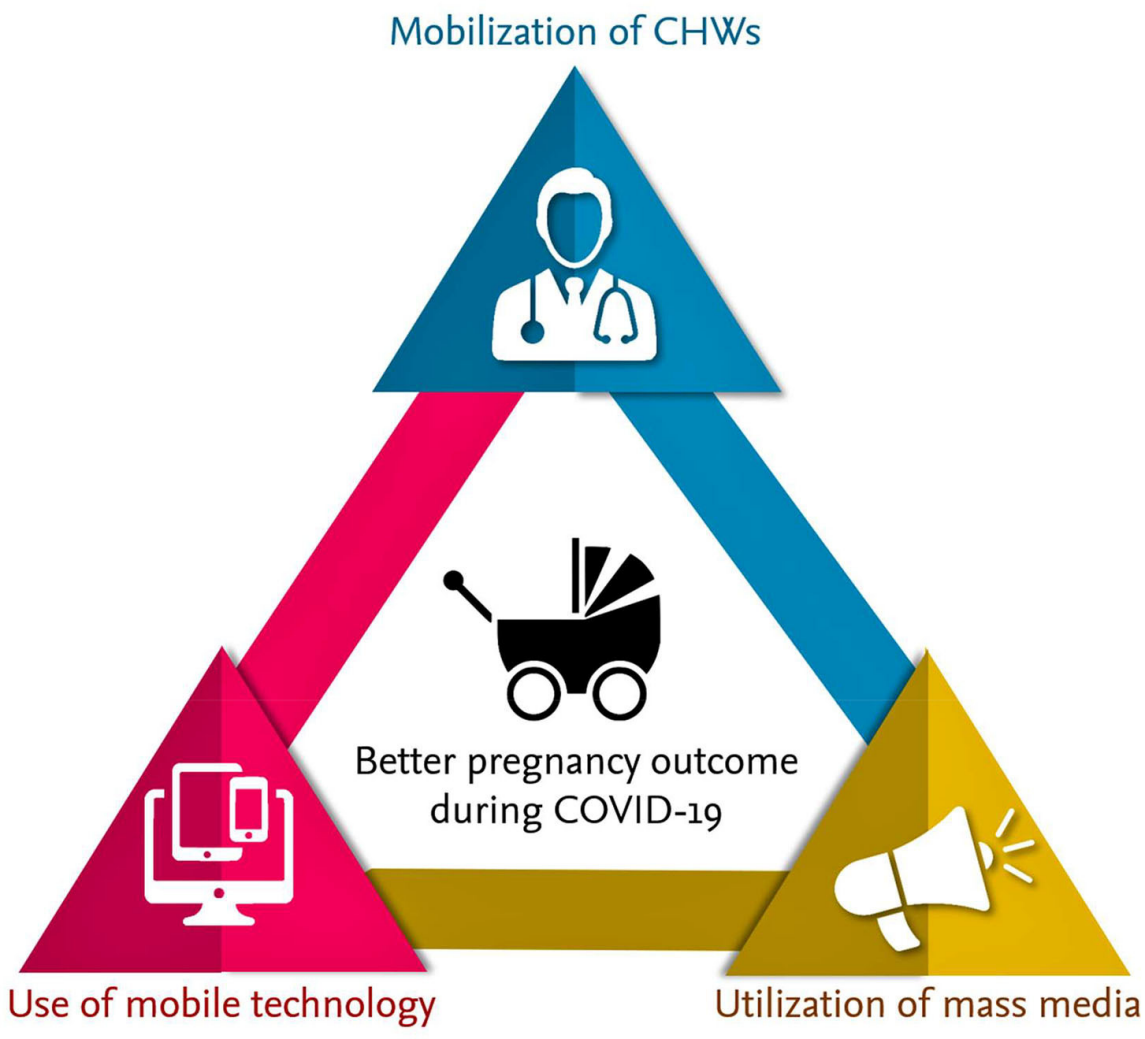
Involvement of CHWs, utilizing mobile healthcare service, and taking advantage of mass media communication on identifying the danger signs during pregnancy could partly mitigate the complications to minimize fatalities.

According to 2016 WHO reports, an estimated 303,000 women died from pregnancy-related complications and within the first month of life, around 2.7 million newborns died. Among these deaths, 2.6 million were stillborn. Studies show that providing quality health care during pregnancy and childbirth can prevent many of these deaths. Globally, around 64% of women receive ANC services (28, 29). The WHO's new ANC model increases the number of contacts with the healthcare providers throughout the pregnancy from four to eight visits. A higher frequency of contacts with healthcare providers is associated with a reduced likelihood of stillbirths. This is because of the early detection and management of potential pregnancy-related complications. The WHO has proposed a minimum of eight contacts for ANC; such an increased number of contacts can decrease perinatal deaths by up to 8 per 1,000 births when compared to a minimum of four visits (29, 30). Currently, many countries are progressively adopting the new model of eight ANC to improve the health of the pregnant mother and fetus. Nevertheless, confinement measures may hinder women from attending ANC as per schedule, and alternative measures must be considered. During this COVID-19 pandemic, WHO recommended six in-person visits and two virtual visits (3rd and 4th) to reduce the number of times the patient needs to travel and attend hospital/clinics. Using strategies like the involvement of CHWs, utilizing mobile healthcare service, and taking advantage of mass media communication on identifying the danger signs during pregnancy could partly mitigate the challenge (Figure 2). Less in-person visits and more online consultations are used in many places to provide ANC during the ongoing COVID-19 pandemic, with encouraging feedback from both care recipients and providers. However, further research using randomized control trials is needed to determine the online ANC delivery system's overall pregnancy outcomes. The cost and benefit analysis of online ANC service is also required to assess the feasibility of continuing online ANC service by the decision-making authorities (31). Of relevance, analysis of mobile health's cost-effectiveness for ANC and facility births showed that mobile health programs were relatively inexpensive and saved lives (for the dollar investment) in Nigeria (32).

## Priority Action Steps

In some developing countries like Rwanda, CHWs are trusted frontline health personnel. CHWs are members of the communities where they work and they are usually selected by the communities, answerable to the communities for their activities as well as being supported by the healthcare system (33). In Bangladesh, CHWs are appointed by the government as family welfare assistants, and some are trained as traditional birth attendants for performing safe delivery. The third Global Forum on Human Resources for Health in 2013 concluded that CHWs and other frontlines primary health care workers are the essential workforce to achieve the goals of universal health care and recommended for their integration into the national health systems. Although CHWs are involved in various social works in the community, they are also involved in providing maternal and neonatal healthcare and are trained to follow up women during pregnancy and post-delivery periods. CHWs are trained on identifying danger signs during pregnancy, including APH, eclampsia, hypertension, and malaria, and timely referral to appropriate hospital/clinics so that pregnancy-related complications could be minimized in earlier stages. During this COVID-19 pandemic, these CHWs should be given additional training, not only to report early signs, but also to provide first aid to save the lives due to complicated pregnancies. Who will cater to the training cost and provide protective gear to the CHWs are unsettled issues and need a public-private cost-sharing fusion program. In the case of home deliveries, CHWs should be given the training to manage unexpected post-partum hemorrhage (PPH), and such training should be a priority during the COVID-19 pandemic. Necessary routine testing kits, from pregnancy test to blood sugar test, should be available to these CHWs. Providing adequate training and equipping them with essential materials are important to support pregnant women during COVID-19 pandemic. Also, in the COVID-19 pandemic, providing post-partum care after childbirth should be the continuation of ANC, with virtual support and guidance from the healthcare providers. Additional care should be provided to reduce post-partum depression. Moreover, throughout the pregnancy, adequate nutrition to pregnant women should be ensured for both maternal and fetal health and overall health, in general (34–45).

Mobile health can also be useful during the COVID-19 outbreak. Mobile health is “the use of mobile devices and its associated technology for health interventions” (46, 47). Mobile health can help in the capturing and sharing of texts, videos, audio, and images. A Kenyan study found that mobile health could significantly enhance the treatment of malaria at the remote locations; mobile health was beneficial in the clinical diagnosis as well as management of the disease (46, 48). A report in 2016 of mobile use data found that 40.9 per 100 inhabitants in developing countries are active users of mobile phones (46, 49); there, government initiatives of capitalizing the use of mobile phones for enhancing ANC during the COVID-19 pandemic are necessary. Such government initiatives are likely to be beneficial for developing countries. For the patient's safety and to ensure the delivery of quality care, a standardized protocol for telemedicine must be established by the health regulatory authorities. Since many people in developing countries have access to mobile phones, healthcare professionals can use this technology to keep contact with the pregnant woman or the family to provide necessary healthcare-related information. Additional studies are needed to ensure that patient safety is not compromised by mobile health's ANC service. Healthcare providers should maintain a low threshold until the availability of further safety data regarding online healthcare services (50). Mass media communication can also serve as an important vehicle to provide essential information to increase pregnancy-related awareness in order to have better pregnancy outcomes during the COVID-19 pandemic. Community awareness of the obstetric danger signs and seeking early medical care is crucial for better pregnancy outcomes including a reduction of maternal fatalities. Of relevance, misconceptions, superstitions, and seeking help from traditional healers could hinder pregnant women's attitude and attendance to the ANC clinics (51–54). Health education initiatives on the danger signs to pregnant women may mitigate those potential dangers.

In the period of confinement due to the COVID-19 pandemic, everyone is required to stay home to minimize the spread of the virus. Pregnant women are also required to follow similar instructions, which might impair them in receiving adequate ANC. Since mass media has a powerful influence on people's thinking and behavior, in the era of COVID-19, mass media could play an important role to increase health awareness to reduce pregnancy-related complications (55–60). The public reliance on the media (radio, television, social networking sites) offers a unique tool to deliver health-related information and to increase health consciousness (57–61). The mass media could, therefore, play a vital role in informing the community about the obstetric danger signs and possible measures, including advising appropriate places for managing those danger signs in the COVID-19 pandemic.

Generally, the studies show the need for mental health support during pregnancy: psychiatric disorders like depression and anxiety with domestic violence affect the mental health and well-beings of the mother and her child (62). Similarly, coincidental adverse life events like the current pandemic of COVID-19 may also aggravate the situation. During COVID-19 health professionals, patients, and the general population are under psychological stress, translating in fear, anxiety, insomnia, and depression (63). A study conducted on pregnant women during this time of COVID-19 concerning health anxiety and behavioral changes reported that around half of the participants were worried about their health; about 83% reported heightened anxiety (62). A shortage of healthcare providers in China with uncoordinated mental health services for dealing with the psychological crisis during the COVID-19 pandemic has been documented (64). Establishing appropriate strategies to address the mental health status of pregnant women should be a priority during this pandemic; mobile technology could be used to provide psychological support to pregnant women to reduce fear, anxiety, and depression. Pregnant women should also be encouraged to report any form of domestic violence to healthcare professionals.

## Summary

While other mechanisms of mobilizing people for ANC and management of home delivery cases continue, the governments should also consider active engagement with CHWs, train them with the necessary information, and provide them with the required material for the management of home deliveries. One important unresolved issue is how to keep engaging CHWs and motivate them to adopt added workloads during this pandemic. Further studies will define the types of incentives required for CHWs to provide additional community services (65, 66). The optimal utilization of e-health and e-consultations and virtual ANC consultations may reduce pregnancy-related complications, and therefore improve maternal and neonatal health during the COVID-19 outbreak. However, it is necessary to mention that providing online ANC may be impaired by restricted net access and limited availability of mobile electronic devices to pregnant women to receive online instructions and supervision in developing countries (67). During this pandemic stress, additional support to the mental health of pregnant women should be an essential component of ANC. The involvement of family and friends should be encouraged, with adequate precautions to reduce the risk of COVID-19. Of clinical importance, for high-risk conditions during pregnancy, virtual ANC consultations may not yield the best results. Therefore, creating an individual care plan for high-risk pregnancies instead of a virtual approach may improve feto-maternal outcome. To reduce the risk of COVID-19-related infection, pregnant women should be vigilant, keep social distancing, restrict visitors, and frequently wash hands with soap or use 60% alcohol-based hand sanitizer. Finally, providing necessary training to the healthcare providers in infection management, in addition to ante- and post-natal care, should be a clinical priority to efficiently deal with COVID-19 to minimize fatalities (68). Finally, government initiatives, particularly in developing countries, are needed to support pregnant women who need remote ANC during the COVID-19 pandemic by providing access to mobile devices and network services. Furthermore, government regulations require enforcement to ensure pregnant women's privacy while taking advantage of online ANC services.

## Author Contributions

PU: design and wrote. GN and SM: wrote. AH and KN: revised and wrote. MR: conceptualized and wrote. All authors contributed to the article and approved the submitted version.

## Conflict of Interest

The authors declare that the research was conducted in the absence of any commercial or financial relationships that could be construed as a potential conflict of interest.
